# A Retrospective Study on the Incidence of Periprosthetic Fractures Related to Total Hip Arthroplasty and Postoperative Complications During Hospitalization

**DOI:** 10.3390/clinpract15030042

**Published:** 2025-02-21

**Authors:** Victor Niculescu, Delia Carmen Nistor-Cseppento, Sebastian Tirla, Brigitte Osser, Cristina Aur, Diana Mocuta, Gheorghe Ion Popescu, Radu Dan Necula

**Affiliations:** 1 Doctoral School, University of Medicine and Pharmacy “Carol Davila” Bucuresti, 050074 București, Romania; victor.niculescu@drd.umfcd.ro; 2 Doctoral School of Biomedical Sciences, Faculty of Medicine and Pharmacy, University of Oradea, 410087 Oradea, Romania; brigitte.osser@uav.ro; 3 Department of Psycho-Neuroscience and Recovery, Faculty of Medicine and Pharmacy, University of Oradea, 410073 Oradea, Romania; 4 Department of Surgical Disciplines, Faculty of Medicine and Pharmacy, University of Oradea, 410073 Oradea, Romania; d.aur@uoradea.ro (C.A.); dmocuta@uoradea.ro (D.M.); 5 Department 14 Orthopedics-Intensive Care, “Carol Davila” University of Medicine and Pharmacy Bucharest, 050474 București, Romania; gheorghe.popescu@umfcd.ro; 6 Department of Medical and Surgical Specialties, Faculty of Medicine, Transilvania University, 500019 Brasov, Romania; radu.necula@unitbv.ro

**Keywords:** periprosthetic fractures, incidence, associated complication, age, predictor

## Abstract

**Background/Objectives:** Periprosthetic fractures (PFs) are serious complications that can occur after total hip arthroplasty (THA), particularly in elderly patients who often have multiple comorbidities and low bone density. The surgical treatment of PFs typically involves internal fixation or revision arthroplasty, depending on the fracture type categorized by the Vancouver classification. This study examines the annual incidence of PFs and the complications that arise during hospitalization, as well as the predictive role of age in the occurrence of these fractures and their associated complications. **Methods**: Based on a retrospective observational study conducted over three years (2021–2023) at the Bihor County Emergency Hospital in Oradea, we analyzed 783 patients who underwent various hip surgeries. **Results:** The retrospective analysis identified 38 cases of PF out of 768 hip surgeries, resulting in an incidence of PF of 4.5%. Most PFs were classified as Vancouver B, with surgeries mainly involving internal fixation. Complications occurred in 23.68% of cases, including a mortality rate of 7.90%. A correlation analysis examining the relationship between age and post-surgical complications demonstrates a weak and statistically insignificant association (r = 0.120, *p* = 0.478). To highlight whether age is a predictive factor for PFs, we used the linear regression model; this suggests that older age explains 2.7% of the total variability in the incidence of PFs, being statistically significant ([F(1, 766) = 20.923], *p* < 0.001). **Conclusions**: The rising incidence of periprosthetic fractures is closely linked to the increasing number of total hip arthroplasties and the aging population. Fractures of this type are more common in elderly women, with no statistically significant differences have been found between the sexes.

## 1. Introduction

The incidence of periprosthetic fractures (PFs), a serious complication of total hip arthroplasty (THA) [1], increases in parallel with the number of total hip arthroplasty procedures, being more frequent in elderly patients who also present various comorbidities and decreases in bone density. Due to the higher morbidity compared to primary THA [2] and the considerable resources required, they represent a socioeconomic challenge [3]. PFs occur more frequently postoperatively; however, intraoperative occurrence is not excluded (with an incidence of approximately 1.7%) [4].

**Figure 1:**
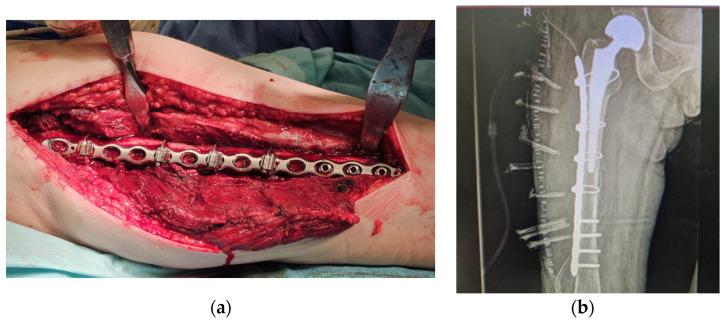
(**a**). Surgical intervention with internal fixation (authors’ archive) (**b**). Periprosthetic fracture fixation—radiological image (authors’ archive).

The standard treatment for PFs consists of surgical intervention, which may include internal fixation or revision arthroplasty with replacement of the femoral component and reduction with cerclage, depending on the Vancouver classification of the fracture type. This is related to the fracture level, the prosthesis’s stability, and the surrounding bone’s quality. If the femoral component is stable, open reduction and internal fixation are recommended (Figure 1a). 

The most common type of PF is Vancouver B. For Vancouver B1-type fractures with a stable implant, open reduction with internal fixation using a plate (Synthes) [5] and cerclage wires is advised (Figure 1b). This method is particularly advantageous given the advanced age of the patients and the presence of comorbidities. If anatomical reduction cannot be achieved, revision arthroplasty may be necessary [6].

The recommended protocol for Vancouver B2 and B3 types involves revising the femoral component with a long uncemented stem. It is important to acknowledge that immediate weight-bearing post-surgery can be a disadvantage, along with issues related to fragment fixation. Research has indicated that uncemented revision arthroplasty tends to be more effective because it avoids the complications associated with cement extrusion [7]. A study conducted by J.L. Maggs and colleagues, published in 2021 (n = 87), suggests that a modification of the Vancouver classification might be needed for cemented arthroplasties, categorizing them as B2W (well-fixed bone cement) or B2L (weakened) [8]. For Vancouver C PFs, treatment that includes the use of allografts is recommended.

Revisions of hip arthroplasty due to periprosthetic fractures account for less than 20% of all total hip revision arthroplasties [7]. These procedures are complex and can result in serious postoperative complications. The incidence of PFs after revision surgery varies between 11% and 30% [4]. Identified risk factors include the type of prosthesis used (cemented vs. non-cemented), the design of the prosthesis, and the surgical approach. Other important factors include the patient’s gender, age, and presence of comorbidities, particularly decreased bone density. Research indicates that cemented prostheses and collared prostheses have a lower risk of periprosthetic fractures [9]. In a study by I.A. Malek and collaborators (2016) involving 448 patients, it was found that the direct anterior approach to surgery is associated with a significantly higher incidence of periprosthetic fractures [10]. Conversely, R.A. Sershon and collaborators (2021), in a larger study involving 6309 total hip arthroplasties (THAs), concluded that the choice between anterior and posterolateral surgical approaches did not significantly impact the occurrence of periprosthetic fractures [11].

Immediate complications reported in the literature include issues at the wound site, whether or not they are associated with infection (either superficial or deep), with an incidence of less than 15%. Other complications include deep vein thrombosis (1.3%) and pulmonary embolism (0.7%). Neurological complications, such as paralysis of the sciatic nerve, may occur in 1.3% of cases. Additional complications that have been reported include prosthesis dislocation (6.0%) and refracture (10.0%) [7]. The risk of developing a deep periprosthetic infection is estimated at 1%, which can lead to serious long-term consequences [12]. Mortality associated with PFs during hospitalization is below 2.5%. However, the mortality risk increases over time, being approximately 3.5% at 30 days, 4.8% at 90 days, and 13.4% at one year, according to data published by J.N. Lamb in 2022 (n = 4841). The incidence of complications is notably higher in elderly patients over 85 years old who often have a reduced functional reserve. There are no reported differences in complications based on the type of intervention performed [13]. These complications associated with PFs after THA involve various controversies and knowledge gaps, particularly in classification, management strategies [14], the timing of intervention, patient factors, outcomes, and long-term consequences. There are inconsistencies in how PF results are reported [15]. There is a lack of consensus on the risk factors that predispose patients to PFs, making it difficult to identify high-risk populations. Addressing these issues and controversies is essential for enhancing the management and outcomes of periprosthetic fractures following hip arthroplasty. This is particularly true given the considerable debate in the existing literature regarding these risk factors for PFs. Therefore, we support the idea proposed by Y. Zhu et al. which advocates efforts to update and conduct quantitative analyses aimed at optimizing the screening of risk factors in patients undergoing THA to help prevent the development of PFs [1].

Starting from the hypothesis that the incidence of PFs associated with THA increases in parallel with the number of THAs and the aging of the population, this study aimed to evaluate (1) the annual incidence of PFs that are subject to surgical treatment; (2) the presence of complications and mortality associated with PFs during hospitalization; (3) age as an associated/predictive factor for PFs and their complications.

## 2. Materials and Methods

### 2.1. Database

**Figure 2:**
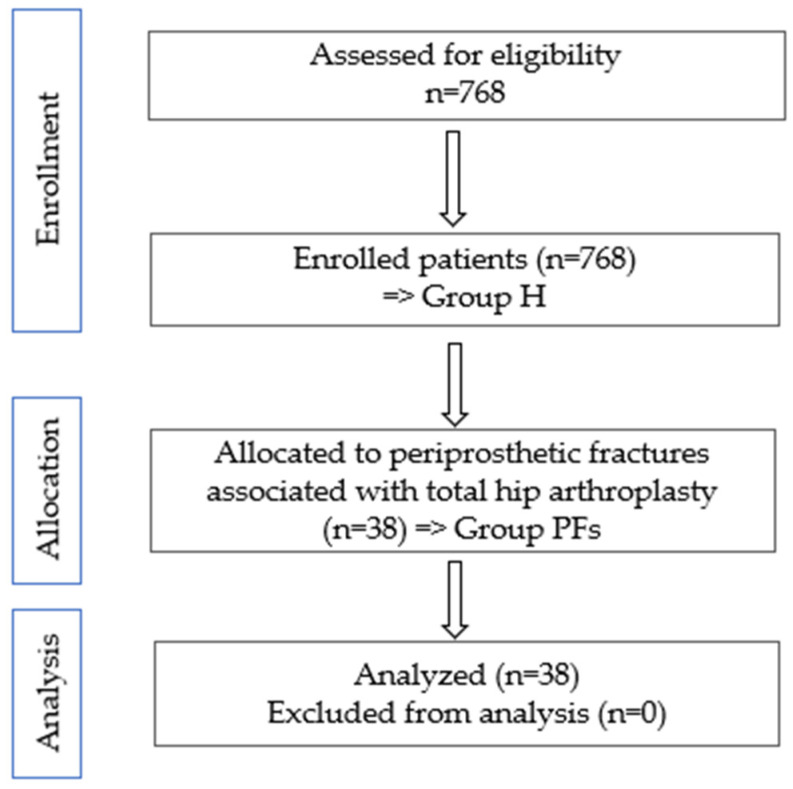
CONSORT flow diagram of study.

A retrospective observational study was conducted from 1 January 2021 to 31 December 2023. Out of a total of 768 patients who underwent surgical interventions at the hip level—primary total hip arthroplasty (THA), total or partial revisions, or surgeries with internal fixation (Group H)—at the Bihor County Emergency Hospital in Oradea (Group H), we selected cases involving periprosthetic fractures associated with THA. To mitigate selection bias, we took steps to ensure rigorous case selection and complete data collection. In total, we identified 38 cases that required therapeutic management for this diagnosis, referred to as Group PFs (Figure 2). 

### 2.2. Study Tools

The evaluation focused on the type of prosthesis, surgical technique, risk factors, comorbidities, complications (as classified by the Clavien–Dindo Classification), and postoperative progress during hospitalization. Out of 38 cases identified by PFs, only 1 periprosthetic fracture occurred in a cemented total arthroplasty. The remaining fractures, which made up 97.36%, occurred in uncemented total arthroplasties (ZIMMER). The surgical approach used was lateral in all cases.

The recruited patients were on specific medications for their associated comorbidities, which included antihypertensives, diuretics, hypoglycemic agents, and anticoagulants (administered postoperatively). They also received antiresorptive drugs, vitamin D, and chondroprotective drugs. For neurological conditions, patients were prescribed specific treatments, including antiepileptics (e.g., gabapentin, n = 10), neurotrophic vitamins (B vitamins, n = 10), antiparkinsonian (n = 2) and psychotropic (n = 2) drugs, and antidepressants (n = 4). All patients were retired and their daily activities mainly involved self-care and housework.

**Figure 3:**
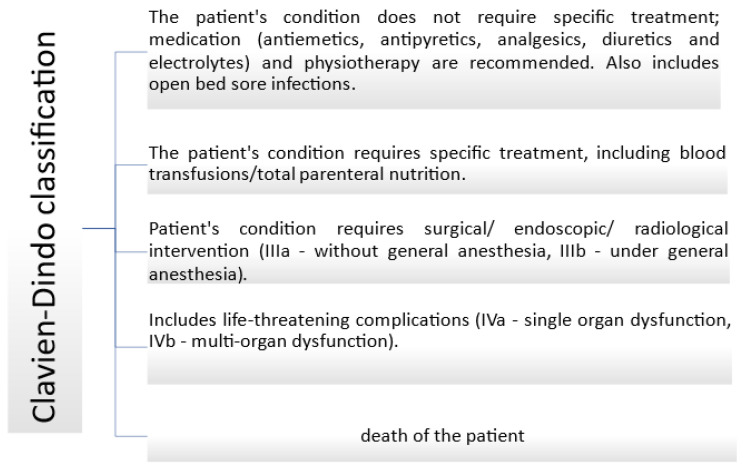
The Clavien–Dindo Classification.

The Clavien–Dindo Classification categorizes postoperative complications based on their severity, particularly those that may threaten life or lead to permanent disability. This classification is divided into five grades, as summarized in Figure 3 [16].

### 2.3. Ethical Approval

Ethics committee approval (No. 35964/21/11/2024) was obtained for this study. The research was conducted in accordance with the World Medical Association Declaration of Helsinki guidelines.

### 2.4. Statistical Analysis

Data processing was performed using JASP version 0.18.1.0. Mean values of parameters, frequency ranges, standard deviations, and statistical significance tests using Student’s method (*t*-test) were used to compare means, and the level of statistical significance was 0.05. Levene’s test was used to assess the homogeneity of dispersion. If the homogeneity of dispersion was not respected between the two groups and the variances were significantly different, the Mann–Whitney U test was used to compare the two groups. To compare other distributions between groups, we used the Chi-square method. A multiple linear regression model was fitted to identify factors influencing the quality of life score.

## 3. Results

### 3.1. The General Characteristics of the Cohort

The general characteristics of the cohort are summarized in Table 1. The participants have an average age of 65.77 years (±10.4), indicating that they are in the process of transitioning into older adulthood. Approximately 75% of the patients are over 60 years old, and more than 50% come from rural areas. The number of hip surgeries increased every year (187/2021–266/2022–315/2023).

The clinical parameters evaluated include high blood pressure (54.4%), chronic kidney disease (55.72%), pulmonary disease (6.38%), chronic venous disease (16.80%), obesity (45.70%), vitamin D deficiency (13.90%), and osteoporosis (44.10%). Among the patients, 95.30% have at least one associated pathology. Of these, 35.50% have two comorbidities, while 40% have more than three. The average number of associated comorbidities in the cohort is 2.26 ± 1.22.

### 3.2. Distribution Based on the Type of Intervention at the Cohort Level

Approximately 95% of patients undergoing hip interventions received primary total hip arthroplasty (Table 2). The total revision of prostheses represents 1.50% of the total number of THAs performed during the evaluated period.

### 3.3. General Characteristics of PF Group

The analysis of the data in Table 3 reveals that the average age of patients with PFs is higher than that of the overall patient cohort. PFs predominantly affect females, with a ratio of 71.05% compared to 50.80% in the total cohort. Additionally, the presence of associated pathologies is consistent with the evaluated cohort, with both groups showing rates exceeding 95%.

**Figure 4:**
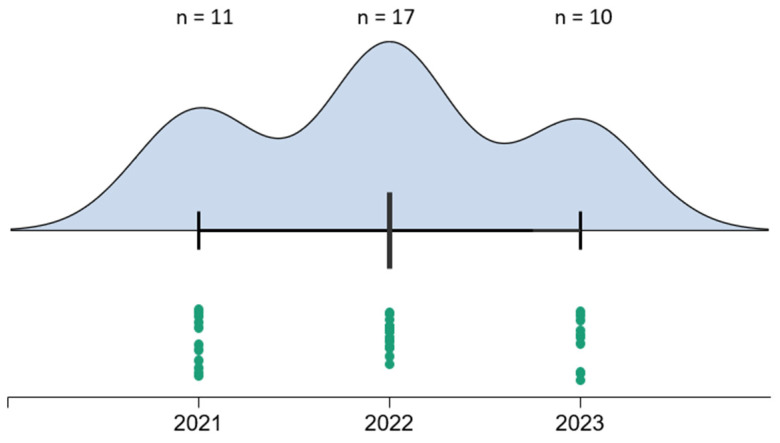
Annual distribution of PFs.

The mean value of age (Figure 4) in women is higher vs. the mean value of age of men (70.33 ± 13.88 vs. 65.91 ± 10.95) but without a significant difference (*p* = 0.35).

### 3.4. Frequency of PFs by Years of Study

The upward trend in PFs is noted in the period 2021–2022, supported by specialized research [17]. In 2022, the frequency of patients with PFs (17 patients) was higher than in 2021 when the orthopedic department treated 11 cases. In 2023, the number of FP patients decreased to only 10 patients, making it lower compared to both 2021 and 2022. The decline in the number of PFs may be linked to the educational prevention measures implemented within the orthopedic department. This correlation is noteworthy since the type of prosthesis and surgical techniques have remained unchanged in 2023.

### 3.5. Distribution According to the Etiology That Led to THA (PF Group)

The data analysis in Table 4 indicates that degenerative hip pathology is the most frequent reason for total hip arthroplasty (THA), accounting for approximately 70% of cases.

### 3.6. Distribution Based on the Level of Periprosthetic Fracture and the Type of Intervention

Out of a total of 38 patients included in the PF group, 34 benefited from uncemented THA (approximately 90%). Table 5 suggests a high frequency of Vancouver B PFs (over 90%). One patient fell into Vancouver stage A and underwent surgical treatment with cerclage. A percentage of 68.42% underwent treatment with internal fixation and cerclage; modular revision was performed on one patient (2.63%), while total revision was necessary for a percentage of 28.94% (eleven cases).

### 3.7. Distribution According to the Clavien–Dindo Classification

**Figure 5:**
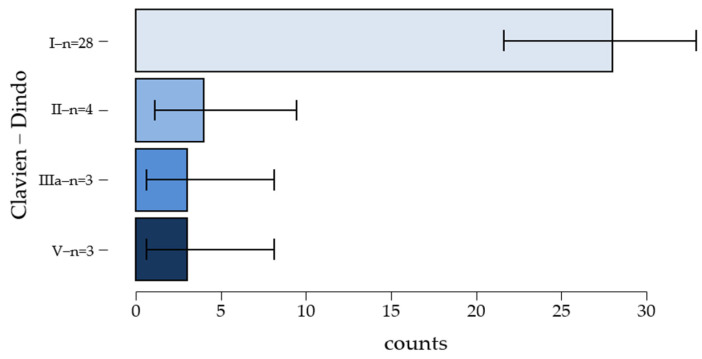
Incidence of immediate surgical complications. Grade I—28 patients; Grade II—4 patients; Grade IIIa—3 patients; Grade V—3 patients.

The analysis of the data in Figure 5 suggests that the majority of patients (92.10%) experienced complications in categories 1, 2, and 3, which did not pose a threat to the patients’ lives. A total of 73.68% experienced category 1 (minor) complications, with the majority being female (n = 21, Table 6); three patients (7.90%) required surgery without anesthesia for debridement due to wound infections, while four patients underwent treatment for phlebitis secondary to the insertion of a peripheral venous catheter (local topical treatments with heparin). However, three patients experienced major complications (massive digestive hemorrhage, acute liver failure, and metabolic acidosis) which led to their deaths.

The frequency of complications in categories 1, 2, and 3 is significantly higher among women than men. However, in category 5 (deaths), the number of recorded deaths among men is twice that of women, with two deaths for men compared to one for women.

### 3.8. Age Differences in the Incidence of Complications

**Figure 6:**
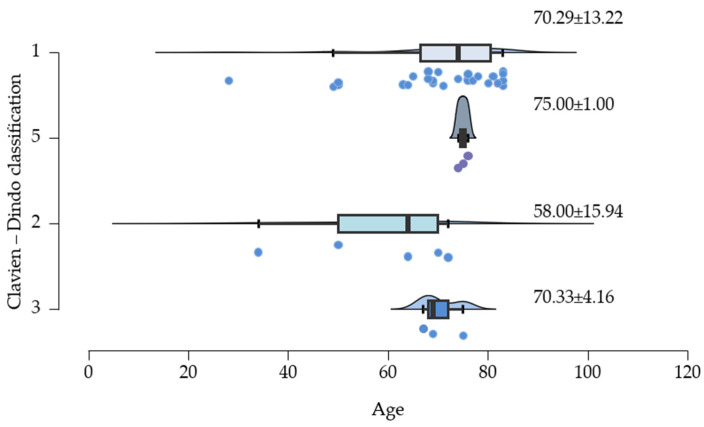
The average ages associated with post-surgical complications.

Figure 6 illustrates the average ages linked to post-surgical complications. It shows that the deaths of the three patients are associated with a mean age of 75 years. Additionally, grade 3 complications are also connected to older age, specifically patients over 70 years old.

### 3.9. Distribution of Complications by Study Year

The contingency table, Table 7, indicates that in 2022, the most serious post-surgical complications occurred, resulting in three deaths; additionally, four patients experienced category 2–3 complications. However, we noted a decrease in the incidence of complications in 2023.

Post hoc analysis reveals significant differences in the categories of postoperative complications between 2022 and 2023 (*p* = 0.027) (Table 8); this outcome is expected due to the increased number of PFs in 2022.

**Figure 7:**
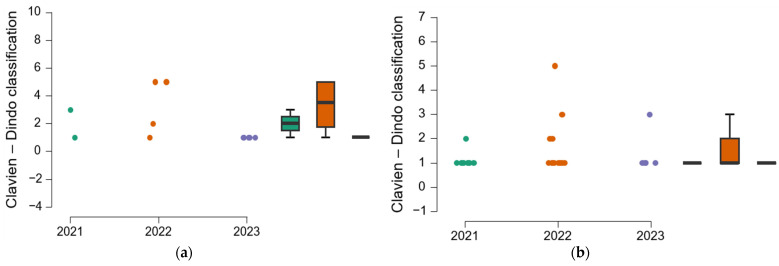
Distribution of complications/year: (**a**) men; (**b**) women.

The data in Figure 7 illustrate the distribution of complications by sex and year of study. Female patients experience the highest number of complications, peaking in 2022, although no statistically significant differences are observed between the two sexes (*p* = 0.093).

### 3.10. Age and Other Associated/Predictive Factors for Periprosthetic Fractures and Their Complications

**Figure 8:**
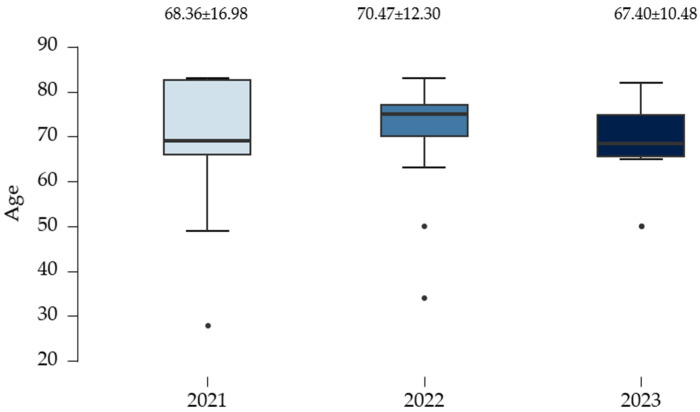
The mean age of patients with PFs/year of study.

The data analysis in Figure 8 indicates that the mean age of the study participants was higher in 2022.

Table 9 indicates that there are no significant differences in mean age and sex between 2021 and 2022 (*p* > 0.05). However, in 2023, the mean age for women is considerably higher than that for men (72.80 ± 6.22 vs. 62.00 ± 11.64). This difference may help explain the higher frequency of complications observed in women.

Levene’s test shows a normal distribution in terms of age. Correlation analysis of age with post-surgical complications shows a weak and statistically insignificant association (r = 0.120, *p* = 0.478).

To highlight whether age is a predictive factor for PFs, we used the linear regression model; this suggests that older age explains 2.7% of the total variability in the incidence of PFs, being statistically significant ([F(1, 766) = 20.923], *p* < 0.001) (Table 10).

We then examined the coefficients of the regression model to determine if age significantly predicts the variability in fractures (Table 11). The results indicate that age is a predictor for these fractures (*p* < 0.001).

## 4. Discussion

In the case of periprosthetic fractures, the challenges that arise are complex. These include issues related to diagnosis, accurate classification, and determining the appropriate therapeutic approach, all of which depend on the fracture level and the stability of the prosthesis [13]. Additionally, functional performance in these patients is diminished due to reduced mobility and the development of muscle hypotrophy [18].

During the study period (2021–2023), a total of 768 hip surgeries were performed. An increasing trend in THA cases was observed, with the numbers rising from 176 to 249 and then to 305. This trend aligns with the increases reported by the Romanian Arthroplasty Register [19]. During the period studied, THAs occupied approximately 95% of hip surgeries. The remaining interventions were modular (0.13%), total (1.43%), and internal fixation (3.38%) revisions.

One of this study’s objectives was to identify the incidence of PFs associated with THA. Analysis of the collected data suggests an incidence of 4.95% (38 PFs out of a total of 768 hip surgeries), compared to the 2.7% incidence reported by L. Zagra et al. [20] in 2021 (38 PFs out of 1390 THAs). Notably, the incidence of PFs in 2021, which was during the COVID-19 pandemic, was lower than that in 2022. This discrepancy may be attributed to population movement restrictions during the pandemic, although L. Zagra et al. did not report significant changes during that period [20]. The findings from this study are comparable to those reported by Toogood et al. in 2015, which indicate a frequency range of 4.2% to 7.4% [21]. PFs were predominantly found among women, comprising over 70% of cases, which is consistent with the results of a previous study by the same authors [13]. Our study indicates a higher prevalence of PFs in females, although no significant gender differences were observed, similar to the findings of Abdel et al. [22]. The average age of patients with PFs was 70.33 ± 13.88 years, a period when the incidence of osteoporosis increases, leading to a higher risk of fractures [23]. Additionally, sarcopenia, which is linked to advanced age due to a decrease in muscle mass and function, further raises the risk of falls [24,25].

The high frequency of PFs classified as Vancouver B is comparable to findings in other studies. However, in contrast to data from the Swedish National Hip Arthroplasty Registry, our study reveals that the incidence of Vancouver B2 PFs was roughly half (21.05% compared to 53%). The gold-standard treatment for Vancouver B2 and B3 PFs is femoral component revision, which is performed in over 85% of cases [7]. In our study, total revision was applied to all cases of Vancouver B3 PFs and four cases classified as Vancouver B2 (50%).

In terms of complications, minor complications were present in 73.68%, while major complications (category 5) were observed in 7.90%. Four patients experienced infection of the wound, representing 10.5%. This suggests a lower incidence compared to the specialized literature, which reports an infection rate of 15%. Phlebitis secondary to peripheral venous catheter insertion occurred at a rate of 7.89%. The risk of developing phlebitis can be decreased by implementing appropriate interventions [26]. Major complications during hospitalization—such as gastrointestinal hemorrhage, metabolic acidosis, and acute liver failure—were encountered in three cases (7.90%), leading to patient deaths. This rate is significantly higher, roughly three times greater than the 2.5% reported by J.N. Lamb et al. in 2022 (n = 4841) [13]. However, it is similar to the mortality rate reported at a six-month follow-up by Young SW in 2008 [27]. All three deaths (two men and one woman) occurred in 2022, with an average patient age of 75 years. Risk factors included cardiovascular diseases, Alzheimer’s disease, and osteoporosis.

The higher mortality determined in our study could be explained by the association with the significant percentage of comorbidities that have a recognized role as risk factors in PFs. The role of comorbidities in favoring the occurrence of PFs is discussed further. More than 97% of the patients with PFs in our study had several associated comorbidities: hypertension (over 75%), varicose veins in the lower limbs (71.05%), diabetes mellitus (60%), chronic kidney disease (84.21%), obesity (68.42%), osteoporosis (23.68%), gonarthrosis (84.21%), and neurological diseases (36.84%). Comorbidities of the cardiovascular system were found to be risk factors for PFs [28]. The retrospective study by B. Redondo-Trasobares et al. (n = 38 patients with PFs of the knee, mean age 72.49 years) aimed to identify risk factors for periprosthetic knee fractures; female gender, dementia, and motor impairment/motor/muscle tone changes in Parkinson’s disease were identified as risk factors. The authors argue the role of these factors by the fact that, taking into account the advanced age of the patients, the effect of estrogen is lost and bone density decreases; thus, the female sex is more prone to periprosthetic fractures [29]. Presbyphagia [30] associated with old age will cause sarcopenia, which increases the risk of falls. Associated neurological diseases further increase the risk of falls. Low bone density, characteristic of old age, is common in patients undergoing THA. Published studies suggest that antiresorptive medication favors an increase in periprosthetic bone mineral density, thus reducing the risk of PFs [31]. A study conducted by D. Maman et al. (2024) analyzed 1,634,615 cases of primary total hip arthroplasty (THA) to compare outcomes between patients with and without periprosthetic fractures. The results indicated that patients with periprosthetic fractures had a higher prevalence of comorbidities. The specific comorbidities found more frequently among these patients included chronic lung disease, hypertension, type 2 diabetes mellitus, and osteoporosis [15]. These comorbidities were also present in a significant percentage of patients included in our study. BMI is an important risk factor for PFs along with the other identified risk factors [31]. Analysis of our study data suggests an increased percentage of obesity (over 60%). The type of anchoring is still under debate. The study published by Assil-Ramin Alimy et al. (2024) suggests that more than 50% of orthopedic surgeons prefer cement anchorage; especially in patients with low bone density, cementing would have a protective role [32], reducing the incidence of PFs [33]. In our study, 97.4% of patients had an uncemented hip replacement. A clear explanation for the decrease in the incidence of PFs in 2023 has not been identified. Extensive studies are needed to confirm the benefits of patient education regarding fracture prevention in patients with THA (performed at the orthopedic department) and osteoporosis screening. In our study, the number of patients receiving antiresorptive treatment was higher in 2023 compared to 2022 (5 patients vs. 3 patients).

### 4.1. Future Directions

Contemporary strategies for managing periprosthetic fractures focus on both preventive measures and advancements in implant technology. Innovations in prosthesis design aim to enhance load distribution and reduce the risk of osteoporosis-related bone stress, which can compromise bone integrity and increase susceptibility to fractures [34]. The study published by F. Washburn et al. (2023) suggests the importance of coronal valgus alignment. A malalignment above 3 degrees is a risk factor for PFs [31]. These efforts represent a significant advancement in improving clinical outcomes and lowering the incidence of periprosthetic fractures.

### 4.2. The Strengths and Limitations of This Study

In our opinion, the length of this study is a strength. This study, to our knowledge, proves to be the first of its kind conducted in Romania. However, this study also has some limitations. The retrospective nature of this study inherently introduces some patient selection bias. Also, the study was conducted at a single center and involved a relatively small number of cases; therefore, the results may not apply to a larger population.

## 5. Conclusions

This study indicates that the incidence of these fractures fluctuates over time. Minor complications frequently arise during hospitalization. It is concerning that older women are more likely to experience both periprosthetic fractures and complications related to their treatment. Additionally, it is important to recognize that age is a significant predictor of periprosthetic fractures associated with total hip arthroplasty. Given the considerable debate in the existing literature regarding risk factors for PF, we advocate continued efforts to perform quantitative analyses aimed at optimizing risk factor screening in patients undergoing total hip arthroplasty (THA) to help prevent the occurrence of PF. Such studies could provide valuable insights into optimizing femoral preparation techniques during surgery, ultimately aiming to reduce the incidence of fractures in this patient population.

## Figures and Tables

**Table 1:** General characteristics of cohort.

Parameters		Group H (n = 768)
Demographic data	Age, years, mean ± SD	65.77 ± 10.41
	<40 years, N (%)	17 (2.20)
	41–50 years, N (%)	46 (6.00)
	51–60 years, N (%)	135 (17.60)
	61–70 years, N (%)	306 (39.80)
	71–80 years, N (%)	223 (29.00)
	>80 years, N (%)	41 (5.30)
	Urban environment, N (%)	334 (43.50)
	Female, N (%)	390 (50.80)
Frequency by years of study	2021	187 (24.35)
	2022	266 (34.64)
	2023	315 (41.01)
Associated pathology	Without associated pathology, N (%)	36 (4.70)
	High blood pressure, N (%)	418 (54.40)
	Varicose veins in the lower limbs, N (%)	129 (16.80)
	Diabetes mellitus, N (%)	184 (24.00)
	Chronic kidney disease, N (%)	428 (55.72)
	Lung diseases, N (%)	49 (6.38)
	Obesity, N (%)	351 (45.70)
	Osteoporosis, N (%)	339 (44.10)
	Gonarthrosis, N (%)	213 (27.70)
	Neurological diseases, N (%)	53 (6.90)
	Vitamin D deficiency, N (%)	107 (13.93)

Group H—a group of patients with hip surgery; N—number of patients.

**Table 2:** Distribution based on the type of intervention.

Type of Surgery		N (%)
THA	Unilateral intervention	614 (79.95)
	Contralateral hip arthroplasty (second surgery on the same patient)	116 (15.10)
Surgical intervention to treat PFs	Total revision	1 (0.13)
	Modular revision	11 (1.50)
	Internal fixation surgery	26 (3.38)

N—number of patients.

**Table 3:** General characteristics of PF group.

Parameters		Group PFs
Demographic data	Age, years, mean ± SD	69.05 ± 13.11
	<70 years, N (%)	16 (42.10)
	>70 years, N (%)	22 (57.90)
	Urban environment, N (%)	22 (42.10)
	Female, N (%)	27 (71.05)
Associated pathology	Without associated pathology, N (%)	1 (2.63)
	High blood pressure, N (%)	30 (78.94)
	Varicose veins in the lower limbs, N (%)	27 (71.05)
	Diabetes mellitus, N (%)	23 (60.52)
	Chronic kidney disease, N (%)	32 (84.21)
	Lung diseases, N (%)	8 (21.05)
	Obesity, N (%)	26 (68.42)
	Osteoporosis, N (%)	9 (23.68)
	Gonarthrosis, N (%)	32 (84.21)
	Neurological diseases, N (%)	14 (36.84)
	Vitamin D deficiency, N (%)	10 (26.31)

**Table 4:** Distribution according to the etiology that led to THA.

Etiology	N (%)
osteonecrosis of the femoral neck	3 (7.89)
primary coxarthrosis	24 (63.16)
secondary coxarthrosis	3 (7.89)
femoral neck fracture	8 (21.05)

**Table 5:** Distribution of PFs based on Vancouver classification.

Vancouver Classification	N (%)	Internal Fixation + Cerclage/N	Revision M/T N
A	1 (2.63)	1	-
B1	23 (60.52)	21	1/1
B2	8 (21.05)	4	0/4
B3	6 (15.79)	-	0/6

M—modular revision; T—total revision.

**Table 6:** Gender differences in complications.

Classify Clavien–Dindo	Gender		Total
	f	m	
1	21	7	28
2	3	1	4
3	2	1	3
5	1	2	3

f—female; m—male.

**Table 7:** Contingency table with distribution of complications/study year.

Clavien–Dindo Classification	2021	2022	2023	Total
**1**	9	10	9	28
**2**	1	3	0	4
**3**	1	1	1	3
**5**	0	3	0	3

**Table 8:** Post hoc analysis.

Year		Mean Difference	SE	t	p_tukey_
**2021**	2022	−0.877	0.513	−1.710	0.217
	2023	0.356	0.533	0.668	0.784
**2022**	2023	1.233	0.452	2.729	**0.027**

p_tukey_—*p* value.

**Table 9:** Descriptive table of mean age/year/sex.

Sex	Year	N	Mean ± SD	SE	Coefficient of Variation
**f**	2021	9	68.33 ± 18.98	6.329	0.278
	2022	13	70.77 ± 12.59	3.492	0.178
	2023	5	72.80 ± 6.22	2.782	0.085
**m**	2021	2	68.50 ± 0.70	0.500	0.010
	2022	4	69.50 ± 13.10	6.551	0.189
	2023	5	62.00 ± 11.64	5.206	0.188

f—female; m—male.

**Table 10:** Statistics of the regression model for PFs.

Model		Sum of Squares	df	Mean Square	F	*p*
**H_1_**	Regression	2211.026	1	2211.026	20.923	<0.001
	Residual	80,946.787	766	105.675		
	Total	83,157.813	767			

**Table 11:** Statistically significant predictors of PFs.

Model		Unstandardized	Standard Error	Standardized	t	*p*
**H_1_**		2.171	0.048		45.027	<0.001
	Age	−0.003	7.241 × 10^−4^	−0.163	−4.574	**<0.001**

## Data Availability

All study data are available upon request from the first author.
